# Molecular Mechanism for Hepatic Glycerolipid Partitioning of n-6/n-3 Fatty Acid Ratio in an Obese Animal Biomodels

**DOI:** 10.3390/ijms24021576

**Published:** 2023-01-13

**Authors:** Victor A Zammit, Sang-O Park

**Affiliations:** 1 Metabolic Biochemistry, Warwick Medical School, University of Warwick, Coventry CV4 7AL, UK; 2 College of Animal Life Science, Kangwon National University, Chuncheon-si 24341, Republic of Korea

**Keywords:** animal biomodel, glycerolipid, jugular-vein cannula, n-6/n-3, obese

## Abstract

The n-6/n-3 metabolic pathway associated with hepatic glycerolipid portioning plays a key role in preventing obesity. In this nutrition metabolism study, we used in vivo monitoring techniques with 40 obese male Sprague-Dawley strain rats attached with jugular-vein cannula after obesity was induced by a high-fat diet to determine the molecular mechanism associated with hepatic glycerolipid partitioning involving the n-6/n-3 metabolic pathway. Rats were randomly assigned to four groups (10 animals per group), including one control group (CON, n-6/n-3 of 71:1) and three treatment groups (n-6/n-3 of 4:1, 15:1 and 30:1). They were fed with experimental diets for 60 days. Incorporation rates of [^14^C]-labeling lipid into glycerolipid in the liver were 28.87–37.03% in treatment groups fed with diets containing an n-6/n-3 ratio of 4:1, 15:1 and 30:1, which were significantly (*p* < 0.05) lower than that in the CON (40.01%). However, ^14^CO_2_ emission % of absorbed dose showed the opposite trend. It was significantly (*p* < 0.05) higher in a treatment groups (n-6/n-3 of 4:1, 15:1 and 30:1, 30.35–45.08%) than in CON (27.71%). Regarding the metabolic distribution of glycerolipid to blood from livers, phospholipid/total glycerolipid (%) was significantly (*p* < 0.05) lower in CON at 11.04% than in treatment groups at 18.15% to 25.15%. Moreover, ^14^CO_2_/[^14^C]-total glycerolipid (%) was significantly (*p* < 0.05) higher in treatment groups at 44.16–78.50% than in CON at 39.50%. Metabolic distribution of fatty acyl moieties flux for oxidation and glycerolipid synthesis in the liver were significantly (*p* < 0.05) better in order of 4:1 > 15:1 > 30:1 than in the CON. Our data demonstrate that n-6/n-3 of 4:1 could help prevent obesity by controlling the mechanism of hepatic partitioning through oxidation and esterification of glycerolipid in an obese animal biomodel.

## 1. Introduction

The balance of n-6 to n-3 fatty acid ratio (i.e., n-6/n-3) is very important for obesity prevention and human health care. Obesity occurs when triacylglycerol (TAG) is accumulated excessively in white adipose tissues due to excessive food intake and lack of exercise, with increased number and size of adipocyte cells [1,2]. Food intake and energy metabolic regulation are controlled by neuronal signal transduction in the hypothalamus. Disorder of such signal transduction can lead to energy imbalance and obesity [3]. Humans and monogastric animals cannot synthesise n-6 or n-3 fatty acid because they do not have an endogenous enzyme necessary to insert a cis double bond at the n-6 or the n-3 position of a fatty acid. Thus, these essential fatty acids have to be supplied through diet [4,5].

Due to the significant development of the edible oil industry, along with economic and industrial development, the intake of saturated fat and n-6 has increased. Excess n-6, that is, the intake of food with a high n-6/n-3, raises the level of low density lipoprotein-cholesterol (LDL-C) in the blood and causes metabolic diseases, including obesity [5,6]. The n-6/n-3 in western people, due to consumption of foods rich in n-6, is 15–20:1. It is 1:1 for Eskimo in Greenland and 12:1 for Japanese living by the sea. Such high n-6/n-3 has a close relationship with a significant increase in the prevalence of overweight and obesity [7,8,9]. It has been reported that the intake of a diet with a low n-6/n-3 in an obese animal biomodel can lower blood TAG, total cholesterol and LDL-C levels and help prevent metabolic diseases in humans. Although biochemical mechanisms for lipid metabolism of n-6/n-3 in a hyperlipidemic, nutritional monogastric and obese animal biomodel have been reported, hepatic biodistribution of glycerolipid in an obese animal biomodel remains unknown [10,11,12]. SREPBs (SREPB-1α, SREPB-1c, SREPB-2), LPL, PPAR and NF-κB genes are related to obesity by regulating biosynthesis and oxidation of fatty acid [12,13]. Shin et al. [12] found that when n-6/n-3 is decreased from 71:1 to 4:1 in an obese animal biomodel, the blood lipid profile, leptin and insulin levels, SREPBs-mRNA expression in liver and the number of adipocytes are decreased, whereas PPAR and LPL-mRNA expression in adipose tissues are upregulated. Shoelson et al. [14] reported that signaling pathways leading to NF-κB are activated in insulin-responsive tissues of obese and high-fat-diet-fed animals.

Since body tissues use a small amount of fatty acid newly synthesised and secreted from the liver for esterification of glycerol partially, the distribution of fatty acid between oxidation (formation of ketone bodies and generation of CO_2_) and esterification (glycerolipid synthesis) is very important [10,11]. In vivo monitoring can be used to study a living animal by installing a jugular vein-cannula in order to solve an in vitro conundrum (the level of hormone and a very small amount of lipid existing in the body cannot be actually replaced) regarding the biodistribution of fatty acid between different metabolic events in the liver tissue [10,11,12]. In vivo monitoring between oxidation and esterification of glycerolipid related to cholesterol, TAG and phospholipid biosynthesised and secreted from the liver is available for investigating the biodistribution mechanism of lipid metabolism in the body related to obesity. Using in vivo monitoring of an obese animal biomodel enables precise understanding of the hepatic biodistribution mechanism between oxidation and esterification of glycerolipid by n-6/n-3 [10,11,12,13,14]. Almost no relevant report is available currently on the biodistribution mechanism between oxidation and esterification of glycerolipid newly synthesised and secreted from the liver of an obese animal biomodel attached with a jugular-vein cannula (below: an obese animal biomodel) based on investigation through in vivo monitoring. Thus, the objective of this study was to determine the molecular mechanism involved in hepatic glycerolipid biodistribution using an obese animal biomodel (male Sprague-Dawley strain rats attached with a jugular vein-cannula and induced by a high-fat diet).

## 2. Results

### 2.1. Incorporation Rate of ^14^C-Labeling Lipid and ^14^CO_2_ Emission Rate into Glycerolipid

The incorporation rate of ^14^C-labeling lipid (hereinafter, ^14^C) and the ^14^CO_2_ emission rate into glycerolipid in the blood and livers after supplying a diet containing n-6/n-3 in an obese animal biomodel are shown in Table 1. The incorporation rate of ^3^H-labeling lipid (hereinafter, ^3^H) in the liver was significantly higher than the incorporation of [^14^C] in the liver and plasma (*p* < 0.05). The incorporation rate of [^3^H] in the liver ranged from 94.52% to 95.60%, which was very high. However, the incorporation rate into the blood was between 1.10% and 1.12%, which was very low. There was no significant difference in the incorporation rate of [^3^H] into the liver and blood between treatment groups. The incorporation rate of [^14^C] into the liver and blood was higher in the order of 71:1, 30:1, 15:1 and 4:1, according to n-6/n-3. The range of the incorporation rate was 28.87–40.01% in the liver and 17.20–23.70% in the blood. In addition, the incorporation rate in the liver was higher (*p* < 0.05) than that in the blood. [^14^C]-cholesteryloleate is metabolised into [^14^C] oleic acid and cholesterol in the liver. Production of ^14^CO_2_ according to glycerolipid metabolism was higher in the order of 4:1, 15:1, 30:1 and 71:1, according to n-6/n-3. It showed significant (*p* < 0.05) differences between treatment groups. Production of ^14^CO_2_ ranged from 9.78% to 25.25%, indicating that glycerolipid metabolism was carried out faster in animals after ingesting a diet that contained lower n-6/n-3 (for example, in the group with an n-6/n-3 of 4:1) (Table 1).

### 2.2. Ememission of ^14^CO_2_ and Accumulation rate of ^14^C-Labeling Lipid in Tissue

The emission of ^14^CO_2_ and the accumulation rate of ^14^C-labeling lipid in tissues were investigated after supplying a diet containing n-6/n-3 in an obese animal biomodel. Data are shown in Table 2. There were significant (*p* < 0.05) differences in the [^14^C] absorption rate and ^14^CO_2_ emission between treatment groups. The accumulation rates of [^14^C] in adipose, liver and muscular tissue were lower (*p* < 0.05) in the order of 4:1, 15:1 and 30:1 in treatment groups, compared to the rate in the control group (n-6/n-3 of 71:1). The emission rate of ^14^CO_2_ was higher (*p* < 0.05) in the order of 4:1, 15:1 and 30:1 in treatment groups than that in the control group with n-6/n-3 of 71:1. The accumulation rate of [^14^C] in the adipose tissue of the control group with dietary n-6/n-3 of 71:1 was greater than that in the 4:1, 15:1 and 30:1 treatment groups by 71.14%, 69.15% and 5.97%, respectively. Muscle is known to be an important part of the removal of cyclic TAG for oxidation. The control group with dietary n-6/n-3 of 71:1 had the highest accumulation rate of [^14^C] in the hind-leg muscle, which was greater (*p* < 0.05) than that in the 4:1, 15:1 and 30:1 treatment groups by 99.72%, 41.98% and 7.41%, respectively (Table 2).

### 2.3. The Ratio of Cholesteryl Ester Metabolized by the Liver

The ratio of cholesteryl ester metabolised by the liver collected from the esterification product of the blood and liver was investigated after supplying a diet containing n-6/n-3 in the obese animal biomodel. The data are shown in Table 3.

The cholesteryl ^14^C-oleate % of total glycerolipid metabolised in the liver was lower in the 4:1, 15:1 and 30:1 groups, compared to that in the control group with dietary n-6/n-3 of 71:1. It showed significant differences among treatment groups (*p* < 0.05). The amount of TAG secretion was lower in the treatment groups (in the order of 4:1, 15:1 and 30:1 for n-6/n-3) than in the control group with n-6/n-3 of 71:1. It showed significant differences among treatment groups (*p* < 0.05). The distribution rate of phospholipid for total glycerolipid was higher in the treatment groups (in the order of 4:1, 15:1 and 30:1 for n-6/n-3) than in the control group with n-6/n-3 of 71:1. It showed significant differences among treatment groups (*p* < 0.05). Production of ^14^CO_2_ for [^14^C]-total glycerolipid was higher in the treatment groups (in the order of 4:1, 15:1 and 30:1 for n-6/n-3) than in the control group with n-6/n-3 of 71:1. It showed significant differences among treatment groups (*p* < 0.05). The distribution rate of glycerolipid secreted to the blood from newly injected [^14^C] in the liver was lower in the order of 4:1, 15:1, 30:1 and 71:1 in the groups for n-6/n-3 in the case of TAG. However, the secretion rate of phospholipid and ^14^CO_2_ production increased (Table 3).

### 2.4. The Contribution Level of Glycerolipid within the Metabolic Turning Point

**Figure 1:**
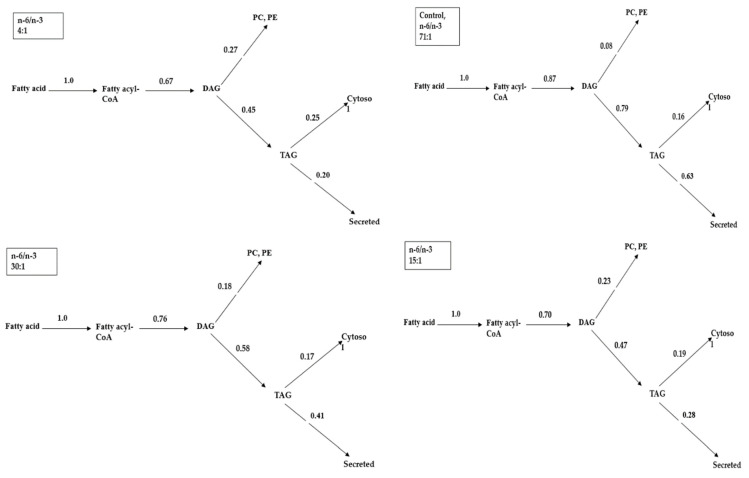
Metabolic distribution of acyl moieties flux for oxidation and glycerolipid synthesis in the liver of obese animal biomodel rat-fed diets containing different n-6/n-3 (*n* = 6). DAG, diacylglycerol; TAG, triacylglycerol; PC, phosphatidylcholine; PE, phosphatidylethanolamine.

The contribution level of glycerolipid within the metabolic turning point was investigated after supplying a diet containing n-6/n-3 in an obese animal biomodel. The results are shown in Figure 1. The secretion rates of [^14^C]-TAG from the liver evaluated from the biodistribution data of in vivo monitoring for the 4:1, 15:1, 30:1, and 71:1 groups were 72.99%, 75.93%, 78.12% and 82.25%, respectively, indicating that TAG biodistribution increased as n-6/n-3 increased. The phospholipid distribution rates in the 4:1, 15:1, 30:1 and 71:1 groups were 25.15%, 18.87%, 18.15% and 11.04%, respectively, indicating that TAG biodistribution decreased as n-6/n-3 increased. In particular, the group with the lowest n-6/n-3 (4:1) had the largest decrease in the secretion of TAG newly synthesised by the liver, with the highest increase of phospholipid.

## 3. Discussion

The reason for using [^3^H] and [^14^C] at the same time for studying the mechanism of metabolic biodistribution of fatty acid in the liver is as follows (Table 1). [^3^H]-cholesteryl oleoyl ether is used in order to correct the lipid metabolism that occurs when [^14^C] is injected, that is, the metabolic rate of very low density lipoprotein-triacylglycerol (VLDL-TAG) synthesised and secreted from the liver [10,11,12,15]. In such cases, the incorporation rate of [^3^H] in the liver is significantly higher than the incorporation rate of [^14^C] in the liver when lipid metabolism is carried out. However, the incorporation rate of [^3^H] into the blood is significantly low or very little. Unlike [^14^C]-cholesteryl oleate, [^3^H]-cholesteryl oleoyl ether does not pass through the above metabolic pathway in the liver. In fact, more than 90% of [^3^H]-cholesteryl oleoyl ether remains in the liver (Table 1). Thus, it is possible to measure the biodistribution mechanism of fatty acids in the liver more accurately using these two isotope indicators [10,11,12,16]. Oleic acid is used for the synthesis of VLDL-TAG through oxidation and esterification. Newly synthesised VLDL travels to the blood 30 min later. Therefore, it is possible to measure the amount of newly synthesised VLDL-TAG secretion into the blood using [^14^C]-cholesteryl oleate [17,18].

In vivo monitoring studies regarding the metabolism of fatty acids in the liver showed a slight difference in ^14^CO_2_ emission kinetics (Table 1). This might be due to direct ^14^CO_2_ generation in the liver and oxidation of [^14^C] ketone bodies in epithelial tissues [10,11,12,19]. Theoretically, [^14^C] ketone bodies can be incorporated into [^14^C]-TAG in lipogenesis tissues. However, it has been reported that its incorporation amount is less than 2% in 1 h after injecting [^14^C] indicator. Therefore, in vivo monitoring studies on ^14^CO_2_ production are more important than studies on the oxidation of [^14^C] ketone bodies [17,20]. The incorporation rate of glycerolipid into the liver and blood from newly injected [^14^C] in an animal biomodel related to lipid metabolism was the highest when n-6/n-3 was 71:1 and the lowest when n-6/n-3 was 4:1, while the result for ^14^CO_2_ production was the opposite [10,11,12], consistent with the results of the present study.

To correct differences in the ^14^CO_2_ emission and [^14^C] absorption rate occurring in the metabolic process after the intake of a chow diet and to present more accurate data, it is advisable to state ^14^CO_2_ emission and the accumulation rate of [^14^C] in tissues in % of absorption rate [19]. These results might be due to an increase in the accumulation rate of lipids at n-6/n-3 of 71:1. This shows a flow of newly injected [^14^C] for accumulating lipids in the adipose tissue rather than oxidation (liver, muscle, brown fat) (Table 2). Muscle is known to be an important part of the removal of cyclic TAG for oxidation. In an obese animal biomodel, n-6/n-3 of 4:1 reduced the accumulation of lipids in tissues by inhibiting the absorption of lipids and by accelerating oxidation at the same time [10,11,12]. Park and Zammit [11] have reported that accumulation rates of [^14^C] in adipose, liver and muscle tissue are lower, but ^14^CO_2_ emission is higher in the groups treated with n-6/n-3 of 71:1, 4:1, 15:1 and 30:1 than that in the control group in a normal animal biomodel attached with a jugular-vein cannula, supporting our findings.

Table 3 shows the concentration (%) of the metabolised amount based on the [^3^H] recovery rate (Table 1) to correct the difference between different measurements according to lipid metabolism. The results of Table 3 indicate that a diet containing n-6/n-3 of 4:1 in an obese animal biomodel can accelerate the oxidation rate of fatty acids. A new finding was that in a hyperlipidemic, nutritional monogastric and obese animal biomodel, n-6/n-3 of 4:1 lowered the secretion to blood from newly synthesised TAG in the liver and simultaneously increased phospholipid secretion and ^14^CO_2_ emission. These results were similar to previous reports showing that lower n-6/n-3 can prevent hyperlipidemia by lowering harmful lipids in the blood [10,11,12].

In Figure 1, the group with the lowest n-6/n-3 of 4:1 had the largest decrease in the secretion of TAG newly synthesised by the liver and the highest increase of phospholipid. Such results come from multiple reduction effects in the direction from biodistribution of acyl moiety to biodistribution of fatty acyl-CoA, diacylglycerol (DAG), phospholipid and TAG [21,22,23,24]. Therefore, an increase in the accumulation of TAG in the cell due to fatty acyl-CoA increase and secretion in the direction of oxidation creates the same contribution level. Synthesis of glycerolipid is the main route of fatty acid metabolism in the liver of humans and animals. The tissue uses a very small amount of newly synthesised fatty acid for partial esterification of glycerol. The biodistribution of fatty acid in the liver between the formation of acylcarnitine for oxidation of fat and esterification for lipogenesis decides the oxidation rate of the fatty acid. Thus, the biodistribution of fatty acid in the liver between oxidation and esterification is very important [10,11,12,19,20,25]. Metabolism of fatty acids in an animal biomodel ingesting a chow diet is carried out in the direction of synthesis of TAG and phospholipid. VLDL containing TAG synthesised by the liver and secreted into the blood is used as the lipid of lipoprotein lipase in adipose tissues of animals [21,26,27]. A new fact indicates that as n-6/n-3 decreased to 4:1 in an obese animal biomodel, an anti-obesity effect was obtained due to the metabolic biodistribution mechanism that decreased the amount of TAG newly synthesised and secreted by the liver. Increased phospholipid at the same time was discovered from these results. Higher n-6 and n-6/n-3 (example: Western diets, n-6/n-3 10–16:1) are associated with obesity via increased white adipose tissue, chronic inflammation and synthesis of proinflammatory cytokine interleukin 6 (IL-6). On the contrary, lower n-6/n-3 can prevent obesity. According to the WHO, an ideal ratio of n-6/n-3 for human health is 4:1, which is recommended to be named as natural food [28,29,30].

In conclusion, lower n-6/n-3 in an obese animal biomodel, especially n-6/n-3 of 4:1, increased CO_2_ emissions and the metabolic biodistribution rate of phospholipid. Although ^14^C-labelled TAG was newly synthesised in the liver of an obese animal biomodel-fed diet containing n-6/n-3, the secretion rate of ^14^C-labelled TAG was lowered. Our data showed that the incorporation of [^14^C] labeling lipid in the liver and plasma after injection of LPS labelled with cholesteryl [^14^C]-oleate, tissue [^14^C] lipid accumulation, and secreted TAG were high, with an increase of n-6/n-3 (4:1, 15:1, 30:1, 71:1) in an obese animal biomodel. However, secreted phospholipid and ^14^CO_2_ emission rates were the opposite. After quantifying the partitioning of flux of fatty acyl CoA, lower n-6/n-3 improved esterification and oxidation of glycerolipid. These data indicate that n-6/n-3 of 4:1 can reduce blood-harmful lipids and prevent obesity by the mechanism of hepatic biodistribution through oxidation and esterification of glycerolipid, which can move lipids from the liver to the blood. Our results suggest that n-6/n-3 of 4:1 in a diet can serve as an ideal ratio for preventing obesity associated with lipid metabolism.

## 4. Materials and Methods

### 4.1. Animal and Experimental Design

Scientific and ethical regulations presented in the EEC Directive (86/609/EEC) were followed for all animal experiment procedures. Animal experiments were carried out after receiving approval from the Institutional Animal Care and Use Committee (IACUC) of Kangwon National University (Approved No. KNU-17102). Forty male Sprague-Dawley strain rats (average body weight: 180–182 g) were purchased from Daehan Bio Link Co., Ltd. (Eumseong-gun, Chungcheongnam-do, Republic of Korea). After one week of environmental adaptation using a chow pellet diet, a high fat diet (HFD, D12451, rodent diet 45 kcal% fat, research diet, New Brunswick, NJ, USA) containing beef tallow [12,30] was then supplied to animals for 42 days to induce obesity [12,30,31]. A total of 40 male Sprague-Dawley strain rats were fed HFD for 42 days to produce obese animals. The obese animal biomodel was produced by attaching a jugular vein-cannula. Experimental animals were randomly assigned to four groups (10 animals per group), including one control group (CON, n-6/n-3 of 71:1) and three treatment groups (n-6/n-3 of 4:1, 15:1 and 30:1) [12,31]. After feeding an experimental diet containing different n-6/n-3 for 60 days, in vivo monitoring was carried out to investigate the biodistribution of glycerolipid in obese animal biomodels.

### 4.2. Experimental Diets and Feeding Management

Purified pellet diet was adjusted to satisfy the requirement of nutrition for rats presented by the American Institute of Nutrition (AIN). It was used as a basal diet. The purified pellet diet contained casein (20%), corn starch (39.8%), sugar (10%), maltodextrin (13.2%), fats (7.0%), α-cellulose (5.0%), AIN-93G mineral mixture (3.5%), AIN-93G vitamin mixture (1.0%), L-cystine (3.0%), choline bitartrate (0.25%) and t-butylhydroquinone (0.0014%). For n-6/n-3 as diet fat, coconut oil (7.0%) was added for the control group (CON, n-6/n-3 of 71:1). The n-6/n-3 was adjusted to 4:1 (perilla oil 1.30% plus corn oil 5.70%), 15:1 (corn oil 6.70% plus perilla oil 0.3%) and 30:1 (corn oil 6.20% plus soy oil 0.80%), respectively, for the three treatment groups [11,12]. An obese animal biomodel was raised in plastic cages at room temperature (22 °C) and humidity of 55% with a 12 h light-dark cycle. The animal biomodel had free access to drinking water and experimental diet ad libitum.

### 4.3. Jugular-Vein Cannulation

**Figure 2:**
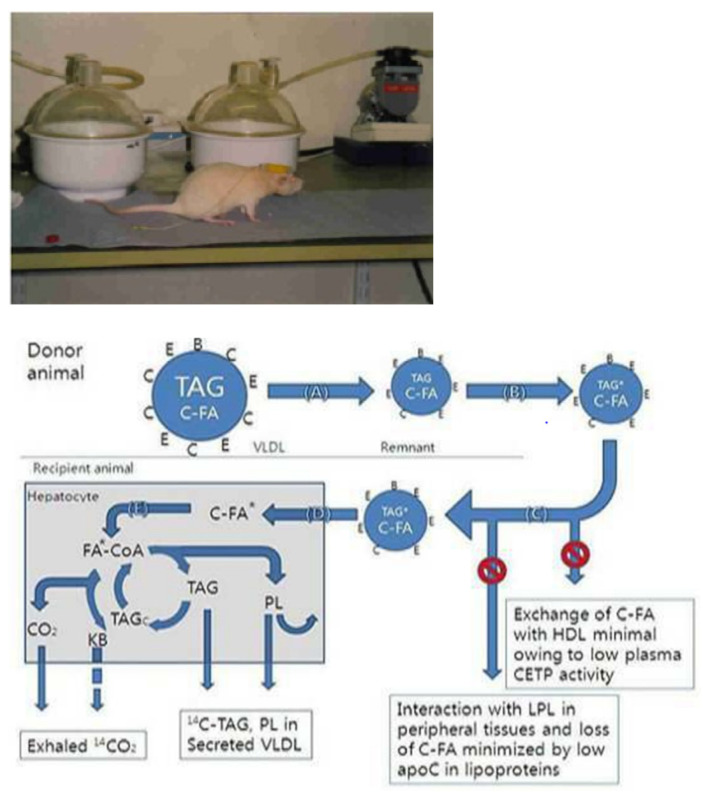
In vivo monitoring technique to metabolic distribution of glycerolipid in obesity animal biomodels. * Indicate the metabolic pathway of cholesteryl-[^14^C]-oleate which is breakdown from TAG* (TAG newly synthesed by cholesteryl-[^14^C]-oleate) in the hepatocyte. A–E indicated Apolipoproteins.

After the feeding trial for 60 days, three animals from each treatment group (a total of 12 animals from four treatment groups) were selected and in vivo monitored after attaching a jugular vein cannula. Hepatic biodistribution mechanism between oxidation and esterification of glycerolipid was investigated by performing in vivo monitoring, as described previously (Figure 2). Jugular-vein cannulation was conducted according to the Europe Laboratory Animal Handling License (SCT-W94058, UK) acquired by the author. Intraperitoneal injection of 0.15 mL mixture of Ketamine (50 mg/mL) and Rompun at 3:1 ratio per 100 g body weight was applied, and a jugular-vein cannula was attached to each animal biomodel. A recovery period of 5 days was given [11,32].

### 4.4. Preparation of Lipopolysaccharides

A 10% fructose solution was supplied to Sprague-Dawley strain male rats weighing 600 g as donor animals to accelerate the secretion of VLDL-TAG newly synthesised by the liver. Lipoprotein isotope labeling was performed using a 10 mL plasma obtained from the abdominal aorta of donor animals after anesthetizing such animals. In other words, after culturing blood VLDL remnants obtained from donor animals, intermediate density lipoprotein (IDL) was separated, and lipoprotein solution (LPS as ApoC-poor remnant lipoproteins, density < 1.015) was produced and labelled with cholesteryl-[^14^C]-oleate and [^3^H]-cholesteroyl ether, as described previously [10,11]. [^3^H]-cholesteroyl ether and cholesteryl-[^14^C]-oleate (Amershm International, Bucks, UK) were used as labeling lipids. These labeling lipids, cholesterol ester transfer protein and reaction reagents were then added to test tubes, cultured and labelled for lipoprotein to produce LPS. LPS was filtered using a filter (pore size: 0.45 μm) before use [10,11,29].

### 4.5. In Vivo Injection and Respiratory Metabolism Trials

The LPS injection amount for animals was determined with a scintillation counter (Packard 1600TR, Hewlett Packard, Palo Alto, CA, USA). It was adjusted to [^14^C] 300,000 dpm and [^3^H] 300,000 dpm. After injecting LPS (0.25 mL/animal) through the jugular cannula, a respiratory metabolism tool that supplied air (5/min rate) (pump: Masterflex model 7524-50; Cole-Parmer Instrument Co., Ltd. Breath sampling bag: Laboratory for Expiration Biochemistry Nourishment Metabolism Co., Ltd.) was inserted, and oxidation rate was calculated by collecting [^14^C]-CO_2_ (^14^CO_2_) using an alkaline solution (100 mL mixture of ethanolamine and ethylene glycol monomethyl ether 1:2, *v/v*). A 1.0 mL triton WR 1339 solution was injected through the jugular cannula after 15 min. Rats were placed in a desiccator chamber for 60 min. They were then anesthetised using pentobarbitone at 60 mg per kg of body weight [10,11].

### 4.6. Thin-Layer Chromatography

After anesthetizing an animal biomodel with the above method, the abdominal cavity of each animal biomodel was cut open, and 3 mL blood was collected promptly from the abdominal aorta while maintaining body temperature of animals using an infrared ray lamp. Liver, hind-limb muscle and adipose tissues were rapidly frozen and stored using liquid nitrogen until conducting biochemical analysis. Lipids were extracted from each tissue, and lipid fraction was separated using thin-layer chromatography (Merck KGaA, Darmstadt, Germany) [11,33].

### 4.7. Statistical Analysis

Statistical analysis was performed using the SAS package program [34]. With n-6/n-3 as the main factor, comparison between groups was performed using general linear modeling. Duncan’s multiple range test was used to test differences in average values between groups. Results are expressed as mean and standard error of mean (SE) of 10 obese animals. The number of animals used for each test is indicated in the table (n = 9) and figure (n = 3) legend. The null hypothesis significance test was set at *p* < 0.05.

## Figures and Tables

**Table 1:** Emission of ^14^CO_2_ and incorporation of [^3^H], [^14^C] label into liver and plasma at 60 min after injection of LPS (d < 1.015) labelled with cholesteryl [^14^C]-oleate in obese animal biomodel rat-fed diets containing different n-6/n-3.

n-6/n-3	Incorporation of [^3^H], [^14^C] Label (% of Injected Dose)				^14^CO_2_ Emission
	Plasma		Liver		
	[^3^H]	[^14^C]	[^3^H]	[^14^C]	
Control (71:1)	1.10 ± 0.02	23.70 ± 0.95 ^1,a^	95.35 ± 3.63	40.01 ± 1.22 ^a^	9.78 ± 0.33 ^d^
4:1	1.13 ± 0.01	17.20 ± 0.68 ^c^	94.75 ± 3.98	28.87 ± 1.08 ^d^	25.25 ± 0.77 ^a^
15:1	1.12 ± 0.01	17.89 ± 0.72 ^c^	95.60 ± 5.02	31.86 ± 1.02 ^c^	24.77 ± 0.63 ^b^
30:1	1.10 ± 0.01	20.62 ± 0.78 ^b^	94.52 ± 3.78	37.03 ± 1.3 ^b^	13.58 ± 0.55 ^c^

^1^ Mean ± standard error. ^a,b,c,d^ Values within the same line with different superscript are significantly different (*n* = 6, *p* < 0.05).

**Table 2:** Emission of ^14^CO_2_ and tissue accumulation of [^14^C] lipid in obese animal biomodel rat-fed diets containing different n-6/n-3.

n-6/n-3	Absorption of [^14^C] Lipid (%)	^14^CO_2_ Emission (% of Absorbed Dose)	Tissue [^14^C] Lipid Accumulation (% of Absorbed Dose/g of Tissue)		
			Adipose Tissue	Liver	Muscle (Hind Leg)
Control (71:1)	70.15 ± 2.31 ^1,a^	27.71 ± 1.10 ^d^	2.01 ± 0.06 ^a^	1.87 ± 0.06 ^a^	0.81 ± 0.07 ^a^
4:1	57.43 ± 2.02 ^d^	45.08 ± 1.77 ^a^	0.58 ± 0.14 ^b^	0.33 ± 0.01 ^c^	0.22 ± 0.02 ^c^
15:1	60.59 ± 2.48 ^c^	43.77 ± 1.56 ^b^	0.62 ± 0.20 ^b^	0.87 ± 0.09 ^b^	0.47 ± 0.03 ^b^
30:1	68.72 ± 2.61 ^b^	30.35 ± 1.07 ^c^	1.89 ± 0.07 ^a^	0.92 ± 0.10 ^b^	0.75 ± 0.10 ^a^

^1^ Mean ± standard error. ^a,b,c,d^ Values within the same line with different superscript are significantly different (*n* = 6, *p* < 0.05).

**Table 3:** Metabolic distribution of glycerolipid to blood from livers in obese animal biomodel rat-fed diets containing different n-6/n-3.

n-6/n-3	Total Glycerolipid (% of Cholesterol [^14^C]-oleate metabolized in Liver)	% Secreted		Phospholipid/Total Glycerolipid (%)	^14^CO_2_/[^14^C]-Total Glycerolipid (%)
		Phospholipid (% of Total Glycerolipid Labelled)	TAG (% of Total Triacylglycerol Labelled)		
Control (71:1)	78.51 ± 3.01 ^1,a^	8.67 ± 0.33 ^c^	64.58 ± 2.27 ^a^	11.04 ± 0.38 ^c^	39.50 ± 1.41 ^d^
4:1	70.22 ± 2.22 ^c^	17.66 ± 0.61 ^a^	51.26 ± 1.82 ^d^	25.15 ± 0.96 ^a^	78.50 ± 2.61 ^a^
15:1	75.07 ± 2.01 ^b^	14.17 ± 0.52 ^b^	57.01 ± 1.88 ^c^	18.87 ± 0.66 ^b^	72.23 ± 2.75 ^b^
30:1	76.66 ± 2.17 ^b^	13.91 ± 0.45 ^b^	59.87 ± 2.01 ^b^	18.15 ± 0.53 ^b^	44.16 ± 1.55 ^c^

^1^ Mean ± standard error. ^a,b,c,d^ Values within the same line with different superscript are significantly different (*n* = 6, *p* < 0.05).

## Data Availability

Data are available on request.
